# Remodeling Lipid Metabolism and Improving Insulin Responsiveness in Human Primary Myotubes

**DOI:** 10.1371/journal.pone.0021068

**Published:** 2011-07-08

**Authors:** Lauren M. Sparks, Cedric Moro, Barbara Ukropcova, Sudip Bajpeyi, Anthony E. Civitarese, Matthew W. Hulver, G. Hege Thoresen, Arild C. Rustan, Steven R. Smith

**Affiliations:** 1 Molecular and Experimental Endocrinology Laboratory, Pennington Biomedical Research Center, Baton Rouge, Louisiana, United States of America; 2 Diabetes Laboratory, Institute of Experimental Endocrinology, Bratislava, Slovak Republic; 3 Department of Metabolic Physiology, Arizona State University, Phoenix, Arizona, United States of America; 4 Department of Human Nutrition, Food and Exercise, Virginia Polytechnic Institute and State University, Blacksburg, Virginia, United States of America; 5 Department of Pharmaceutical Biosciences, School of Pharmacy, University of Oslo, Oslo, Norway; Instituto de Química - Universidade de São Paulo, Brazil

## Abstract

**Objective:**

Disturbances in lipid metabolism are strongly associated with insulin resistance and type 2 diabetes (T2D). We hypothesized that activation of cAMP/PKA and calcium signaling pathways in cultured human myotubes would provide further insight into regulation of lipid storage, lipolysis, lipid oxidation and insulin responsiveness.

**Methods:**

Human myoblasts were isolated from vastus lateralis, purified, cultured and differentiated into myotubes. All cells were incubated with palmitate during differentiation. Treatment cells were pulsed 1 hour each day with forskolin and ionomycin (PFI) during the final 3 days of differentiation to activate the cAMP/PKA and calcium signaling pathways. Control cells were not pulsed (control). Mitochondrial content, ^14^C lipid oxidation and storage were measured, as well as lipolysis and insulin-stimulated glycogen storage. Myotubes were stained for lipids and gene expression measured.

**Results:**

PFI increased oxidation of oleate and palmitate to CO_2_ (p<0.001), isoproterenol-stimulated lipolysis (p = 0.01), triacylglycerol (TAG) storage (p<0.05) and mitochondrial DNA copy number (p = 0.01) and related enzyme activities. Candidate gene and microarray analysis revealed increased expression of genes involved in lipolysis, TAG synthesis and mitochondrial biogenesis. PFI increased the organization of lipid droplets along the myofibrillar apparatus. These changes in lipid metabolism were associated with an increase in insulin-mediated glycogen storage (p<0.001).

**Conclusions:**

Activation of cAMP/PKA and calcium signaling pathways in myotubes induces a remodeling of lipid droplets and functional changes in lipid metabolism. These results provide a novel pharmacological approach to promote lipid metabolism and improve insulin responsiveness in myotubes, which may be of therapeutic importance for obesity and type 2 diabetes.

## Introduction

Insulin resistance is a common feature of obesity, metabolic syndrome and atherosclerosis [1], [2]. Insulin resistance, which can be defined as a state of reduced responsiveness to normal physiological levels of insulin regarding both glucose [3] and fatty acid [4], [5] utilization, plays a major role in the development of type 2 diabetes mellitus (T2DM). Skeletal muscle is critical for glucose disposal *in vivo* [6]. In this respect, skeletal muscle insulin resistance is a strong determinant of T2DM, thus making improvements in insulin responsiveness a nominal feature of its treatment and prevention.

In the last 10 years, a “lipocentric” view of insulin resistance has gained attention. Abnormalities in fatty acid metabolism may result in the inappropriate accumulation of lipids in skeletal muscle, liver, heart and β-cells [7], [8]. This “ectopic” fat deposition, e.g. the accumulation of intramyocellular triacylglycerols (IMTG), has been associated with skeletal muscle insulin resistance (extensively reviewed in [9], [10], [11], [12]). IMTG content is increased in skeletal muscle of sedentary populations including individuals with obesity and T2DM and negatively correlates with insulin-stimulated glucose disposal [13]. An imbalance between free fatty acid (FFA) availability and lipid oxidation is of primary importance in the development of skeletal muscle insulin resistance.

Paradoxically, IMTG content is increased in highly insulin sensitive endurance-trained subjects, suggesting that IMTG *per se* are not causative in skeletal muscle insulin resistance [14]. IMTG content is probably a marker for accumulation of other lipid intermediates (fatty acyl-CoA, diacylglycerol, ceramides and lipid peroxidation products), each with the potential to interfere with insulin signaling and glucose metabolism. These lipid intermediates activate several downstream targets such as PKCθ and the NFκB pathway, thus altering the insulin receptor, insulin receptor substrate 1 (IRS1) and phosphatidyl-inositol-3-phosphate kinase (PI3K) phosphorylation [8], [15] . Therefore, it is proposed that the coupling of lipolysis and lipid uptake to oxidative capacity in endurance-trained individuals limits the production of toxic lipid intermediates and explains the obese and endurance-trained paradox in insulin responsiveness. This hypothesis has not yet been fully explored.

The activation of cAMP/PKA and calcium (Ca^2+^) signaling pathways is known to promote mitochondrial biogenesis and increase lipid oxidation capacity *in vivo* [16], [17], [18]. Cell culture systems using L6 myoblasts have been used effectively to study glucose and lipid metabolism [19], [20], and differentiated human primary myotubes retain many of the metabolic characteristics of mature skeletal muscle [21]. In the present study, we hypothesized that activation of cAMP/PKA and Ca^2+^ signaling pathways in human primary myotubes would induce functional changes in lipid metabolism and therefore improve insulin responsiveness. We provide novel evidence that the PFI-induced cAMP/PKA and Ca^2+^ signaling pathways increase lipid oxidation, storage and lipolysis, as well as improve insulin responsiveness in human primary myotubes.

## Materials and Methods

### Skeletal muscle cell culture and treatment rationale

This study was approved by the Ethical Committee of Pennington Biomedical Research Center. The study was conducted according to the Declaration of Helsinki and in accordance with the Medical Research Involving Human Participants Act. The general principles of informed consent, ethics review and data management were in line with GCP. All participants gave written and verbal consent to all study procedures.

Samples of *vastus lateralis* from four young lean healthy insulin sensitive male subjects (age 28.0±4.0 yrs, BMI 23.5±1.6 kg.m^−2^, glucose disposal rate 12.6±0.7 mg.min^−1^.kg^−1^ as measured by a euglycemic-hyperinsulinemic clamp) were obtained by muscle biopsy using the Bergstrom technique [1]. Satellite cells (quiescent mononuclear muscle cells) were isolated by trypsin digestion, pre-plated on an uncoated petri dish for an hour to remove fibroblasts, and subsequently transferred to T-25 collagen–coated flasks in Dulbecco's Minimum Essential Medium (DMEM) supplemented with 16% Fetal Bovine Serum (FBS) and human growth factors. Cells were passaged once, and mononuclear myoblasts were immunopurified using the mouse monoclonal 5.1H11 anti-CD56 (also known as Neural Cell Adhesion Molecule 1) antibody [2] (Developmental Studies Hybridoma Bank, Iowa City, IA). The recovery of mononuclear myoblasts ranged from 10 to 20% of the total cell count in the preparation. In order to eliminate any genetic or epigenetic influence on the treatment responses, cells from all four donors were pooled. Cells were grown at 37°C in a humidified atmosphere of 5% CO_2_. Differentiation of myoblasts into myotubes was initiated at approximately 90% confluence, by switching to α-MEM with antibiotics, 2% FBS and fetuin for maximal adhesion and growth [3]. The media was changed every other day. During differentiation, cells were treated continuously with 30 µM of palmitate (Sigma-Aldrich, St Louis, MO) and not pulsed (control), or pulsed one hour per day for the last three days of differentiation with a cocktail containing 4 µM of forskolin (Calbiochem Corp., Darmstadt, Germany) and 0.5 µM of ionomycin (Sigma-Aldrich, St Louis, MO) (PFI), to activate cAMP/PKA and Ca^2+^ signaling pathways, respectively. Forskolin (a PKA activator) and ionomycin (a calcium ionophore), known to activate signaling pathways involved in response to exercise (adrenergic and calcium signaling), are used here in pulse to mimic a bout of exercise. Palmitate was coupled to BSA and then dissolved in the media. The 30 uM palmitate concentration used in our experiments is rather low and thus does not induce insulin resistance. Much higher concentrations of palmitate (up to 400 uM) seem to be necessary to induce insulin resistance in myotubes [6]. Pilot experiments for cellular toxicity determined the optimal timing, duration and concentration of palmitate, forskolin and ionomycin (data not shown). Previous evidence [4], [5], as well as our own preliminary data, has shown that lower concentrations of palmitate are capable of increasing fat oxidative capacity of muscle cells. This also explains its additive effect in combination with the other two compounds. We did not measure the PKA activity nor the activity of calcium-activated kinases; nevertheless, based on the available evidence regarding these cAMP- and calcium-stimulated pathways [7], the pulse treatment in given concentrations should have an expected effect on relevant signaling pathways. Previous evidence from our lab also demonstrates the effects of these compounds separately, thus further supporting the additive effect of the combination of the compounds [22]. All experiments were performed after five days of differentiation, when about 70–80% of mononuclear myoblasts had fused to form multinuclear elongated myotubes. All measurements were done in triplicate and across four independent cultures.

### Mitochondrial content and enzyme activity assay

Quantification of mitochondrial content was performed as previously described using mtDNA copy number [23] and Mitotracker Green probe™ (Molecular Probes, Invitrogen, Eugene, OR). The sequences for the primer/probe sets used in TaqMan analysis of mtDNA content for NADH dehydrogenase subunit 1 (ND1) and of nuclear DNA for lipoprotein lipase (LPL) were designed using Primer Express version 2.1 (Applied Biosystems-Roche, Branchburg, NJ) (Table S1). Mitotracker Green probe preferentially accumulates in mitochondria regardless of the mitochondrial membrane potential and thus provides an accurate assessment of mitochondrial mass [24]. Briefly, cells were washed with 1× PBS and incubated at 37°C for 30 minutes with 100 nM Mitotracker Green. Cells were then harvested using trypsin/EDTA and re-suspended in 1× PBS. Fluorescence intensity was detected with excitation and emission wavelengths of 490 and 516 nm, respectively, and values corrected for total protein (mg/ml). β-hydroxyacyl-CoA dehydrogenase (β-HAD) and citrate synthase (CS) activities were determined spectrophotometrically from cell homogenates as previously described [25].

### Lipid oxidation

Cells were pre-incubated for three hours with [1-^14^C] palmitate (1 µCi/ml; PerkinElmer, Boston, MA) and [1-^14^C] oleate (1 µCi/ml; PerkinElmer, Boston, MA) and respective non-labeled palmitate and oleate (100 µM). Palmitate and oleate were coupled to a fatty acid free BSA in a molar ratio of 5∶1. Following incubation, ^14^CO_2_ and ^14^ASM (acid soluble metabolites) were measured as previously described [26]. Briefly, assayed media were transferred to a custom made Teflon 48-well CO_2_ trapping plate that was clamped and sealed. 70% perchloric acid was injected through the perforations in the lid directly into the media, which drives CO_2_ through the tunnel into an adjacent well where it was trapped in 1N NaOH. Following CO_2_ trapping, the media were spun twice and ^14^ASM measured by scintillation counting. Aliquots of NaOH and media were transferred into scintillation vials, and radioactivity was measured on a multipurpose scintillation counter (LS 6500; Beckman Coulter, Brea, CA). All assays were performed in triplicates, and data were normalized to protein content. The ratio of CO_2_∶ASM represents “efficient” lipid oxidation, namely the ratio of lipid completely oxidized to CO_2_ relative to those metabolites of the lipid that are not completely oxidized to CO_2_, in this case ASM.

### Lipid synthesis

At the end of the lipid oxidation assay, cells were washed twice with 1× PBS and harvested into 0.25 ml of 0.05% SDS for subsequent protein measurement and total lipid extraction with 1 ml of chloroform/methanol (2v/1v). Lipids were washed with 70% ethanol and re-dissolved for thin layer chromatography (TLC) (Anal Tech TLC plates, Whatman Ltd., Kent, ME) and run in a mobile phase containing hexane/diethyl ether/acetic acid, v/v/v, 80∶20∶1). Bands corresponding to phospholipids (PL), diacylglycerol (DAG), and triacylglycerol (TAG) were scanned and quantified using Win-Scan Version 3.12 software (Bioscan Inc., Washington, DC).

### Glucose oxidation

Cells were pre-incubated with a glucose- and serum-free media for 90 minutes, followed by three hour incubation with D[U-^14^C] glucose (1 µCi/ml; PerkinElmer, Boston, MA) and 5.5 mM of non-labeled glucose. After ^14^C glucose incubation, ^14^CO_2_ was measured as described above in the “Lipid oxidation” methods section.

### Glucose uptake

Cells were grown and differentiated in 12-well plates. Experimental conditions were as follows: 1 pmol/l, 1 nmol/l, and 1 µmol/l insulin. Before determining glucose uptake, all cell cultures were exposed to basal medium for 1 h. Wells were rapidly washed three times at room temperature with reaction buffer: 150 mmol/l NaCl, 5 mmol/l KCl, 1.2 mmol/l MgSO_4_, 1.2 mmol/l NaH_2_PO_4_, 10 mmol/l HEPES, and 0.1% BSA, pH 7.4, and then incubated in the same buffer supplemented with 2-deoxyglucose (DOG)/2-[^3^H]DOG (10 µmol/l final concentration, 0.2 µCi/well). For the determination of nonspecific DOG uptake, control cultures were incubated with phloretin (100 µg/ml). The reactions were stopped after 15 min by aspirating the reaction mixture and rapidly rinsing each well four times with PBS at 4°C. Cells were solubilized by the addition of 0.5 ml of 0.1 mol/l NaOH. An aliquot of 50 µl was removed for protein determination. The remaining fluid was placed in 5 mL of scintillation fluid. Glucose transport activity is expressed as picomoles of DOG taken up per minute per milligram of total protein.

### Glycogen synthesis

Cells were exposed to DMEM supplemented with [2-^3^H] deoxyglucose (1 µCi/ml, PerkinElmer) and 500 µM non-labeled glucose for four hours and with D[U-^14^C] glucose (1 µCi/ml; PerkinElmer) for four hours in the absence or presence of 100 nM of insulin (Humulin, Eli Lilly, Indianapolis, IN) to study basal and insulin-mediated glycogen, respectively, as previously described [27]. Briefly, after rinsing each well twice with 1× PBS at 4°C, the cells were solubilized by the addition of 250 µl of 30% potassium hydroxide (KOH). The samples were added to 35 µl of 60 mg/ml glycogen (Sigma-Aldrich, St Louis, MO) in distilled water and heated at 70°C for 20 minutes. Following incubation, 1 ml of ice-cold 100% ethanol was added to precipitate glycogen. The tubes were centrifuged (2000 *g* for 20 minutes at 4°C), and the supernatant was immediately removed and discarded. After one wash with 70% ethanol, the glycogen precipitate was re-suspended in 500 µl distilled water, dissolved under shaking for 20 minutes and counted by liquid scintillation (LS 6500; Beckman Coulter, Brea, CA).

### Lipolysis assay

Lipolysis was performed as previously described [28]. Briefly, at day five of differentiation, cells were pre-incubated in serum-free media for 90 minutes. The lipolysis assay was performed over three hours by adding 0.25 ml of Hank's Balanced Salt Solution (HBSS)+2% Bovine Serum Albumin (BSA) without or with 1 µM of isoproterenol hydrochloride (a non-selective β-adrenergic agonist, Sigma-Aldrich, St Louis, MO). Fluorescence intensity of glycerol was detected and values corrected for total protein (mg/ml).

### Immunocytochemistry and confocal imaging

Lipid droplets were visualized using Bodipy 493/503 (Invitrogen Molecular Probes, Eugene, OR). Nuclei were stained using DAPI (Invitrogen Molecular Probes, Eugene, OR). Myotubes were stained as previously described [29]. Briefly, myotubes were rinsed three times with 1× PBS, followed by fixing with 10% formalin for one hour and permeabilization with 0.1% saponin for 20 minutes, and then incubated with the primary antibodies, mouse anti-MHC I (Chemicon International Inc., Temecula, CA) and mouse anti-MHC II (GeneTex Inc., San Antonio, TX), for one hour at 25°C. Following three washes with 1× PBS plus 0.05% Tween 20, cells were incubated for one hour at 25°C with secondary antibodies, goat anti-mouse IgG conjugated to Alexa-680 (Invitrogen Molecular Probes, Eugene, OR). Proteins were visualized by confocal microscopy (Zeiss LSM 510 Meta, Thornwood, NY). Images were captured and analyzed using the Image J version 1.37c software (NIH, USA). Negative controls performed with the addition of a non-specific mouse primary IgG did not present a fluorescent signal.

### Real-time qRT-PCR

Total RNA from cultured myotubes was isolated with Trizol reagent (Invitrogen, Carlsbad, CA). The quantity and integrity of the RNA was confirmed with the Agilent 2100 Bioanalyzer (Agilent Technologies, Palo Alto, CA). All primers and probes were designed using Primer Express version 2.1 (Applied Biosystems-Roche, Branchburg, NJ), and sequences are listed in Table S1. Real-time quantitative RT-PCR reactions were performed as one-step reactions in ABI PRISM 7900 (Applied Biosystems) using the following parameters: one cycle of 48°C for 30 minutes, then 95°C for 10 minutes, followed by 40 cycles at 95°C for 15 seconds and 60°C for 1 minute. For all assays performed using Taqman primers and probe, the ribosomal phosphoprotein large P0 gene (RPLP0) was used as internal control. All gene expression data were normalized by dividing the target gene by the internal control.

### Microarray

Near whole genome transcriptome analysis was performed using the IlluminaTM bead based technology and Sentrix Human-6 V2 Expression BeadChip (Part Number: BD-25-113; Illumina Inc., San Diego, CA). Cell culture experiments were performed in quadruplicate with duplicate well RNAs extracted for each experiment. These eight RNA samples were assayed in duplicate for a total of 16 near whole genome transcriptome datasets. Raw data was imported into SAS v9.1 (SAS Inc., Cary, NC) and low quality data, specifically probes not above background or with high background intensities, were excluded based on the Illumina platform scanning software metric “Detection Value” (p<0.05). MA plots were used to visualize the transcriptome data pre- and post- normalization of the data. Normalization of the raw data was accomplished using SAS code written to implement a Quantile Normalization procedure as described previously [25]. The significance of the fold change of the treatment effect was tested for each gene using ANOVA [PROC MIXED in SAS]. Also, two-sided False Discovery Rate (FDR) was calculated for each gene with a cut point at p<0.05. Using the complete hit list, a pathway analysis was generated using PANTHER (www.pantherdb.org) and is presented in Table 1. All microarray work has been posted in its entirety in a MIAME compliant database such as the Gene Expression Omnibus (GEO) [30], or CIBEX [31] as acceptable public repositories.

**Table 1:** Pathway analysis of PFI-induced gene activation in human primary myotubes.

Biological Processes	Up/Down	p value
Protein metabolism and modification	+	<0.0001
Protein phosphorylation	+	<0.0001
Intracellular signaling	+	<0.0001
Carbohydrate metabolism	+	<0.0001
Signal transduction	+	<0.0001
G-protein mediated signaling	−	<0.0001
Lipid, fatty acid and steroid metabolism	+	<0.0001
Stress response	+	<0.0001
Transport	+	<0.0001
Steroid hormone metabolism	−	<0.0001
Serine/Threonine kinase signaling	+	<0.0001
Oxidative phosphorylation	+	0.00011
Electron transport	+	0.00015
Muscle contraction	+	0.00094
Lipid metabolism	+	0.0014
Carbohydrate transport	+	0.0028
Mitochondrial transport	+	0.0038
Calcium-mediated signaling	+	0.026
Fatty acid β-oxidation	+	0.076

Data are expressed as mean ± SEM of 4 separate experiments. + Indicate up-regulation, - down-regulation of the biological processes in response to the PFI treatment.

### Statistical analyses

All statistical analyses were performed using GraphPad Prism 4.0 for Windows (GraphPad Software Inc., San Diego, CA). A two-tailed t-test was performed to determine the effect of the PFI treatment. Since all variables were normally distributed, correlations were performed in a pairwise fashion using the Pearson product moment statistic. All values in figures and tables are presented as mean ± SEM. Statistical significance was set at *p*<0.05.

## Results

### Induction of mitochondrial oxidative capacity

**Figure 1:**
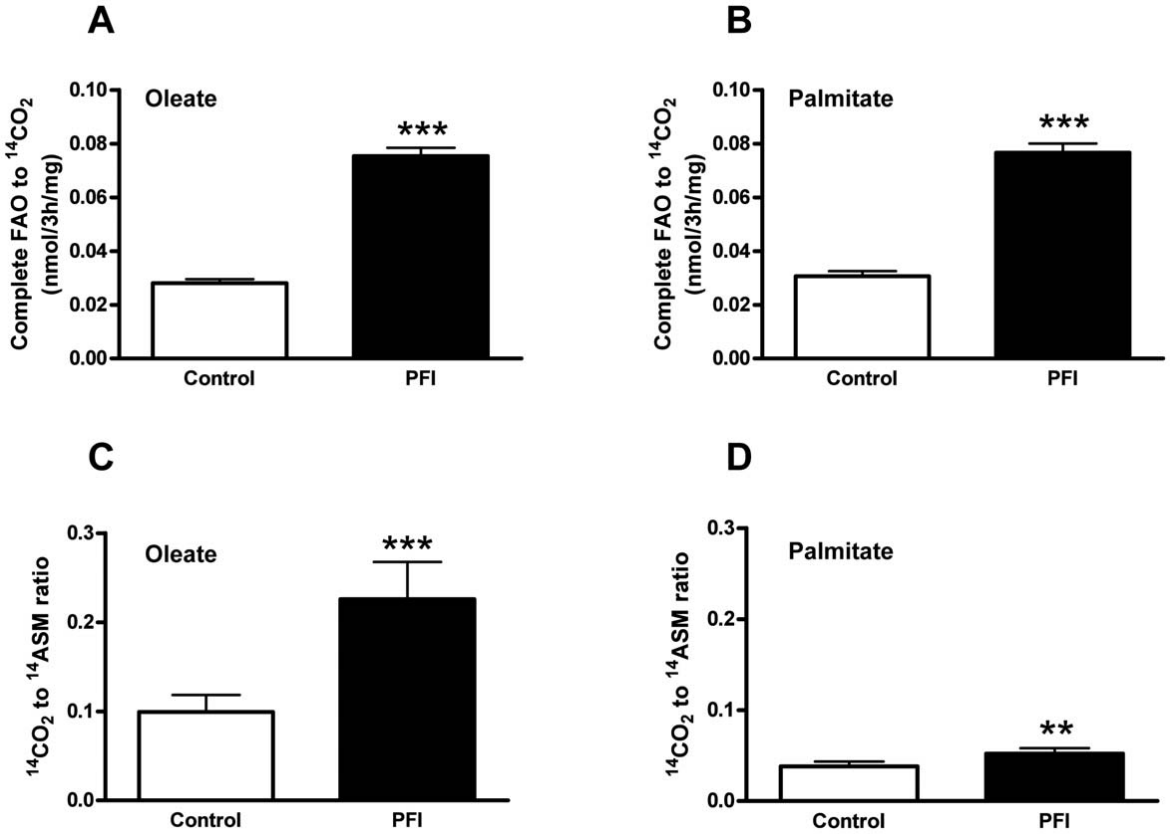
Fatty acid oxidation. Cultured myotubes were incubated with 100 µM of [1-^14^C] oleate (A) or [1-^14^C] palmitate (B) for 3 h, and radiolabel incorporation into CO_2_ was determined as a measure of complete fat oxidation (FAO). Radiolabel incorporation into acid soluble molecules (ASM) was measured to assess incomplete FAO. The ratio of CO_2_ to ASM was calculated for oleate (C) and palmitate (D). Data are expressed as mean ± SEM of 4 separate experiments. ** p<0.01, *** p<0.001 versus control.

**Figure 2:**
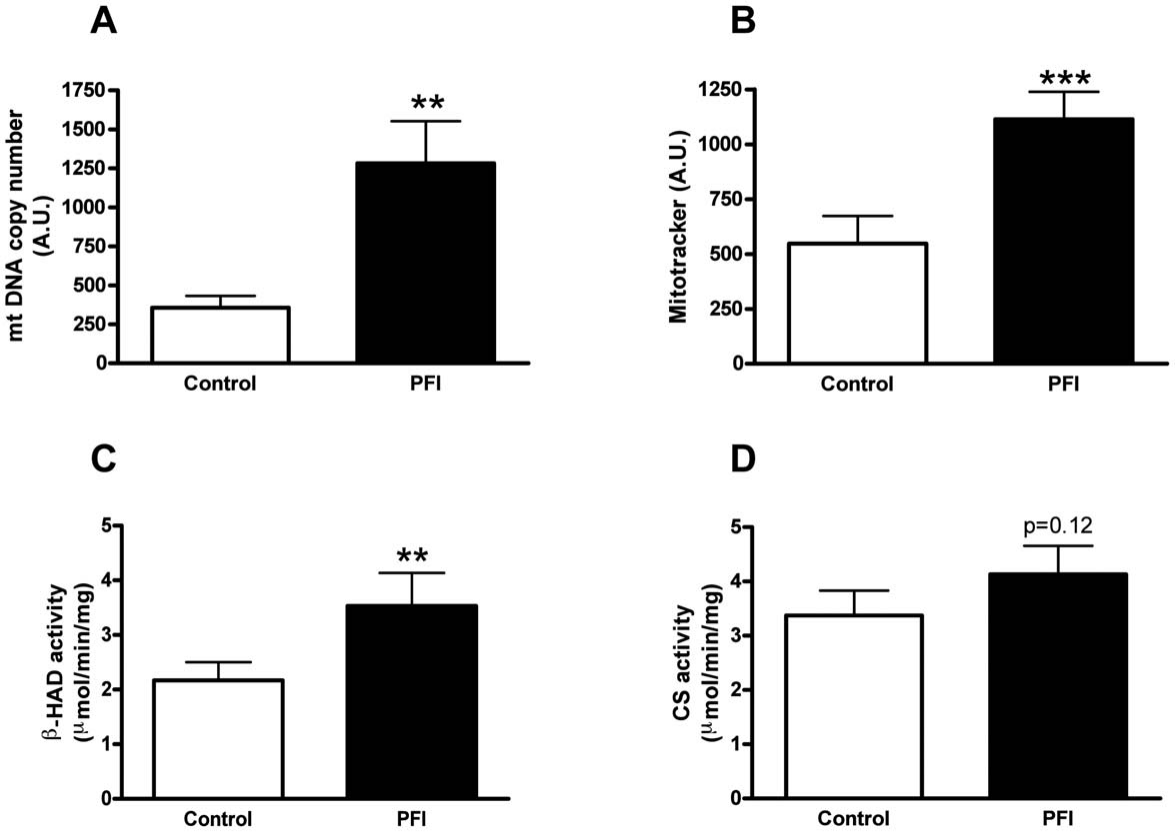
Mitochondrial content and enzymes activity. (A) Mitochondrial content determined by mtDNA copy number. (B) Mitochondrial content determined by Mitotracker Green FM. (C) β-hydroxyacyl-CoA dehydrogenase (β-HAD) activity adjusted for protein content. (D) Citrate synthase (CS) activity adjusted for protein content. Data are expressed as mean ± SEM of 4 separate experiments. ** p<0.01, *** p<0.001 versus control.

The PFI treatment increased the complete oxidation of oleate (2.7 fold, p<0.001) and palmitate (2.5 fold, p<0.001) (Fig. 1A & B) as measured by ^14^CO_2_. Incomplete oxidation as measured by ^14^ASM (acid soluble metabolites) of palmitate (1.074±0.020 vs. 1.703±0.578 nmol/3 h/mg for control and PFI respectively, p<0.001; data not shown) increased after PFI treatment and trended toward a significant increase for oleate (0.417±0.014 vs. 0.457±0.013 nmol/3 h/mg for control and PFI respectively, p = 0.06; data not shown). Interestingly, the ratio of CO_2_ to ASM for oleate (p<0.001; Fig. 1C) and palmitate (p<0.01; Fig. 1D) increased. This ratio is a marker of efficient lipid oxidation in that more CO_2_ is produced compared to ASM. As previously reported, the capacity to completely oxidize oleate to CO_2_ was higher than for palmitate (p<0.001) [32]. Mitochondrial content and mass also increased as indicated by mtDNA (6 fold, p<0.01) (Fig. 2A) and Mitotracker™ Green (2 fold, p<0.001) (Fig. 2B). β-HAD activity increased 1.6 fold (p<0.01); however, the increase in CS activity (1.3 fold) did not reach significance (Fig. 2C & D). Pathway analysis of the microarray gene expression data further revealed an up-regulation of intracellular calcium-signaling (p<0.05), serine/threonine kinase signaling (p<0.0001) and signal transduction pathways (p<0.0001) as expected (Table 1). Interestingly, carbohydrate (p<0.0001) and lipid metabolism (p<0.01) pathways, as well as mitochondrial transport (p<0.01), oxidative phosphorylation (p<0.001) and electron transport chain (p<0.001) pathways, were also up-regulated in response to the PFI treatment (Table 1). Confirming the additive effect of the PFI treatment to mimic exercise, gene expressions involved in muscle contraction were also up-regulated (p<0.001) (Table 1). Activation of cAMP/PKA and Ca^2+^ signaling pathways with the PFI treatment also increased gene expression of two transcription factors involved in the stimulation of mitochondrial biogenesis, PGC-1α and PPARδ by 3- and 2-fold, respectively (Table 2); however, these changes failed to reach statistical significance due to large variation.

**Table 2:** Treatment of human primary myotubes with PFI increases expression of several genes related to mitochondrial biogenesis, lipolysis and lipid storage.

Genes	Fold change	P value
**Mitochondrial biogenesis**		
*PGC-1α*	3.27±0.93	NS
*PPARδ*	2.07±0.45	0.06
**Lipolysis**		
*HSL*	1.47±0.02	<0.001
*ATGL*	1.19±0.08	NS
*CGI-58*	1.81±0.07	<0.01
**Lipid droplet proteins**		
*ADRP*	7.51±2.19	<0.05
*TIP-47*	0.63±0.03	0.02
**Lipid storage**		
*DGAT1*	1.32±0.07	0.01
*GPAT*	1.72±0.23	<0.05
*SREBP-1*	0.60±0.06	<0.01
*FASN*	0.61±0.06	<0.05

Data are expressed as mean ± SEM of 4 separate experiments. NS: not significant. PGC-1α, peroxisome proliferator activated receptor coactivator-1 α; PPARδ, peroxisome proliferator activated receptor δ; HSL, hormone sensitive lipase; ATGL, adipose triglyceride lipase; CGI-58, comparative identification gene 58; ADRP, adipose related differentiation protein; TIP-47, tail interacting protein of 47 kDa; DGAT1, diacylglycerol acyl-transferase 1; GPAT, glycerol-3-phosphate acyl-transferase; SREBP-1, sterol responsive element binding protein-1; FASN, fatty acid synthase.

### Remodeling of lipid droplets

**Figure 3:**
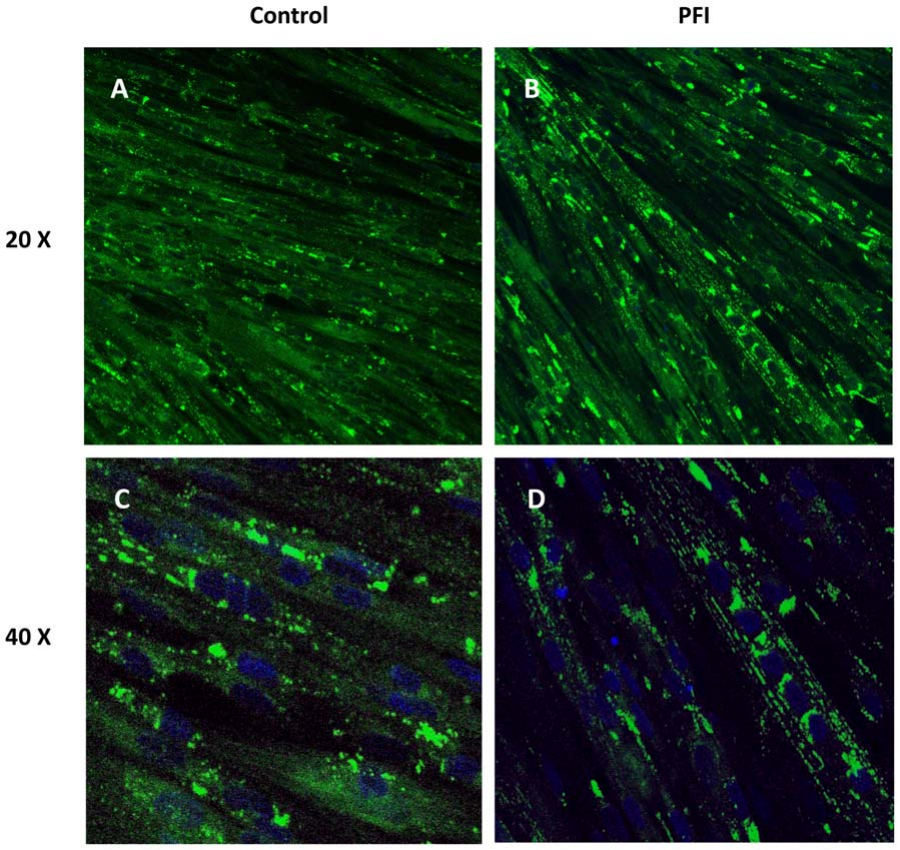
Confocal microscopy of lipid droplets. Lipid droplets were stained with Bodipy 493/503 (green) and nuclei were stained with DAPI (blue). Note the increase in the organization of lipid droplets along the myofibrillar apparatus in panels B (20×) and D (40×) upon PFI treatment.

Confocal microscopy of lipid droplets (stained with Bodipy 493/503) showed a diffuse pattern in lipid staining in control myotubes (Fig. 3A & C). Importantly, PFI treatment caused an increase in the organization of lipid droplets along the myofibrillar apparatus (Fig. 3B), as well as in the perinuclear region as revealed by co-staining of nuclei with DAPI (Fig. 3D). Image quantification revealed an increase in the fluorescence signal in the PFI condition compared to control suggesting an increase in intramyocellular lipid content, together with a redistribution of the lipid droplets. Interestingly, the remodeling of lipid droplets between myofibrils was associated with large changes in the expression of lipid droplet proteins. Two genes associated with lipid droplets were regulated by the PFI treatment. Adipose differentiation related protein (ADRP) gene expression was induced approximately 7-fold (p<0.05) compared to baseline, while tail-interacting protein 47 (TIP47) gene expression was also increased (p = 0.02) (Table 2).

### Functional changes in lipid metabolism

**Figure 4:**
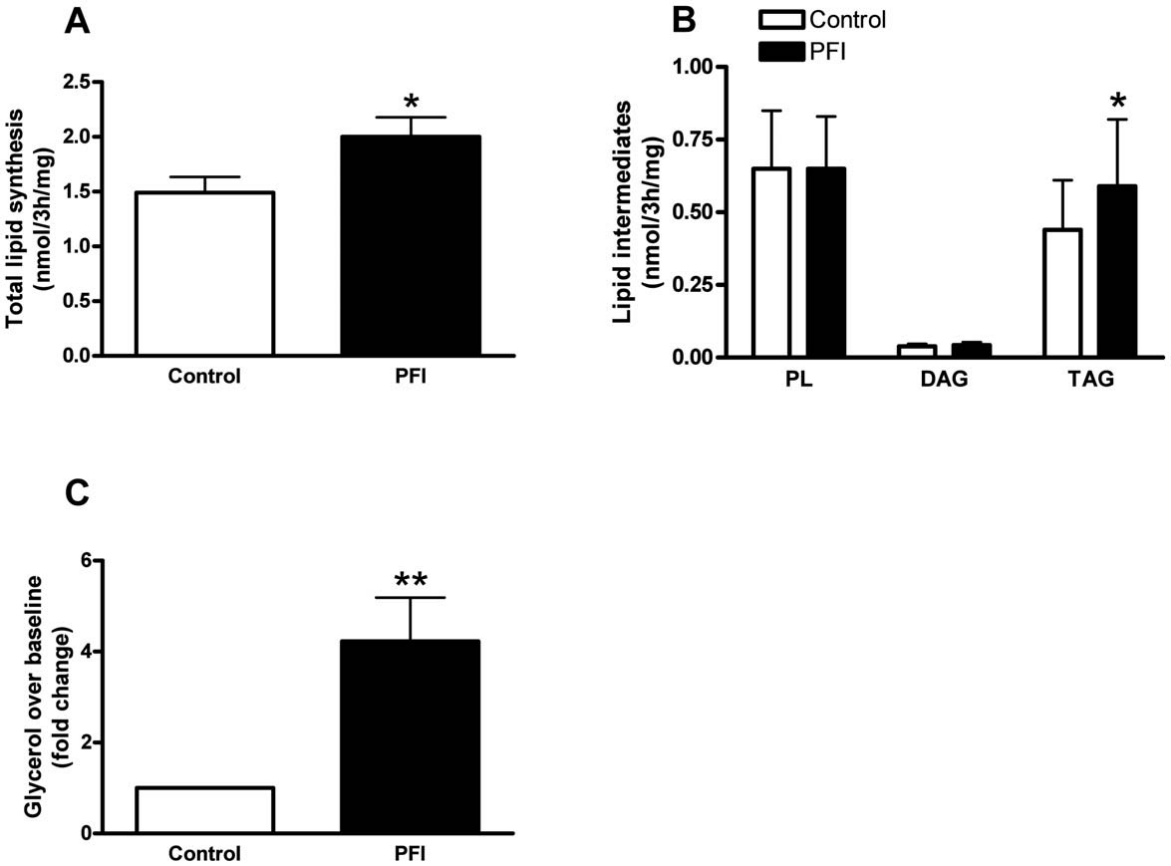
Lipid storage and lipolytic capacity. (A) Cultured myotubes were incubated with 100 µM [1-^14^C] oleate for 3 h, then the cells were harvested and total lipid extracted. Total lipid synthesis was measured as incorporation of radiolabel into total cellular lipids. (B) Lipid intermediates species such as phospholipids (PL), diacylglycerol (DAG), and triacylglycerol (TAG) rates of synthesis were measured by thin-layer chromatography. (C) Lipolysis measured as glycerol release was determined by subtracting baseline values to values during the stimulation with 1 µM of isoproterenol (a non-selective β-adrenergic agonist). The graph represents the changes in lipolysis at baseline in response to isoproterenol in control and PFI conditions. Data are expressed as mean ± SEM of 4 separate experiments. * p<0.05, ** p<0.01 versus control.

In parallel to the remodeling of lipid droplets, we found increased total lipid storage capacity (1.4 fold, p<0.05) in response to oleate during the PFI treatment (Fig. 4A), which is consistent with the increase in intramyocellular neutral lipid content as evidenced above by the increase in the fluorescence signal in the PFI condition compared to control. Interestingly, thin layer chromatography analyses of lipid intermediates species revealed increased triacylglycerol (TAG) synthesis (1.4 fold, p<0.05) after PFI treatment without any changes in diacylglycerol (DAG) and phospholipid (PL) synthesis (Fig. 4B). This increased lipid storage capacity was in line with up-regulation of the glycerolipid biosynthesis rate-limiting enzymes diacylglycerol o-acyltransferase 1 (DGAT1, p = 0.01) and glycerol-3-phosphate acyltransferase (GPAT, p<0.05) gene expression (Table 2). We also observed an up-regulation of sterol regulatory binding protein 1 (SREBP1, p = 0.01) and fatty acid synthase (FASN, p<0.05), key factors in the de novo lipogenesis pathway (Table 2). Lipolytic capacity increased as reflected by a 4-fold increase in isoproterenol-mediated glycerol release (p<0.01) (Fig. 4C). However, basal lipolysis was not different between control and PFI conditions (10.3±1.0 vs. 9.5±1.1 µmol/3 h/mg protein; data not shown). Hormone-sensitive lipase (HSL) and comparative gene identification-58 (CGI-58) gene expression, the rate-limiting enzyme of lipolysis and a potent co-activator of adipose triacylglycerol lipase (ATGL), respectively, were up-regulated (p<0.001 and p<0.01) (Table 2).

### Functional changes in glucose metabolism

**Figure 5:**
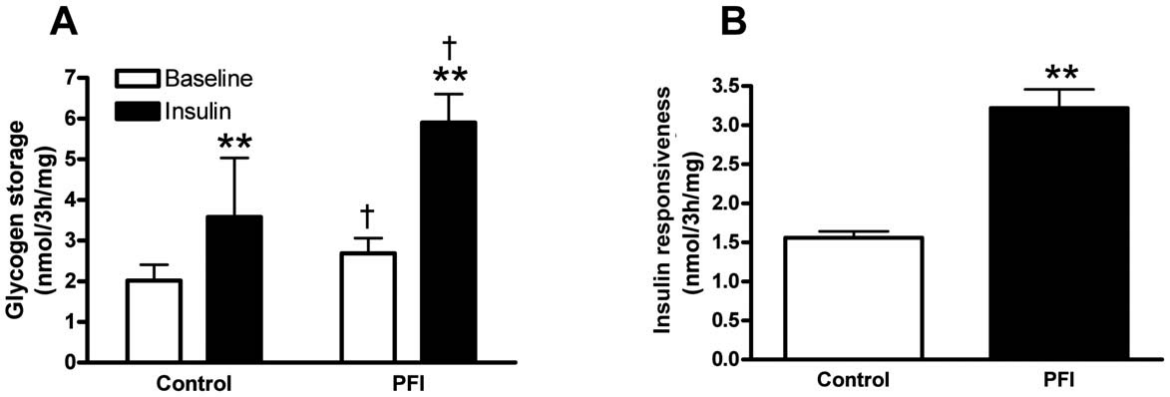
Glucose metabolism and insulin sensitivity. (A) Incorporation of [U-^14^C] glucose into glycogen was measured in either the absence (open bar) or the presence (black bar) of 100 nM of insulin. ** p<0.01 versus baseline, ^†^ p<0.05 versus control. (B) Insulin-stimulated glycogen storage was measured by the change in glycogen storage at baseline in response to insulin and used as a marker of insulin responsiveness in this model. Data are expressed as mean ± SEM of 4 separate experiments. ** p<0.01 versus control.

Basal and insulin-stimulated glucose uptake did not differ between the control and PFI treatment (data not shown). However, baseline (1.3 fold, p<0.01) and maximal insulin-stimulated (2.2 fold, p<0.01) glycogen storage increased after the PFI treatment compared to control (Fig. 5A). The capacity of insulin to promote glycogen storage at baseline was strongly enhanced (2.1 fold, p<0.01) after the PFI treatment (Fig. 5B), indicating an improvement in insulin responsiveness. Additionally, glucose oxidation tended to increase (1.3 fold, p = 0.14) and lactate production tended to decrease (0.5 fold, p = 0.09) after the PFI treatment (data not shown) without reaching significance. These data suggest a shift toward aerobic glucose metabolism. Across experiments, we observed a tight correlation between the increase in mitochondrial oxidative capacity for oleate (R = 0.76, p = 0.004) and palmitate (R = 0.70, p = 0.01) and the increase in insulin responsiveness based on glycogen storage (Table 3). We also found a positive relationship between the increase in insulin responsiveness and the increase in lipid storage (R = 0.73, p = 0.007) and lipolytic capacity (R = 0.78, p = 0.02) (Table 3).

**Table 3:** Correlations between changes in components of lipid metabolism and changes in insulin responsiveness during activation of cAMP/PKA and calcium signaling pathways in cultured human myotubes.

Lipid metabolism components	Insulin-mediated glycogen synthesis (nmol/3 h/mg)	
	R	p
Oleate oxidation (nmol/3 h/mg)	0.76	0.004
Palmitate oxidation (nmol/3 h/mg)	0.70	0.01
Lipid storage (nmol/3 h/mg)	0.73	0.007
Maximal lipolysis (µmol/3 h/mg)	0.78	0.02

The correlations were performed on 12 data points (triplicate wells performed across 4 separate experiments).

## Discussion

Cultured myotubes are a valuable tool for investigation of the interactions between lipid and glucose metabolism. In the present study, we utilized a novel pharmacological treatment to activate cAMP/PKA and Ca^2+^ signaling pathways in primary human myotubes to investigate the impacts on the coordination of lipid metabolism and insulin responsiveness in skeletal muscle. We observed four key structural and functional changes: 1) an increase in mitochondrial biogenesis, 2) an increase in lipid oxidation, 3) a reorganization of lipid droplets and 4) an increase in insulin responsiveness as measured by insulin-mediated glycogen synthesis, comparable to those observed *in vivo*. Furthermore, these results illustrate a tight relationship between oxidative capacity, an organized lipid storage system and insulin responsiveness. This model provides an avenue to enhance our knowledge of the interrelationships of these four key cellular attributes.

Activation of cAMP/PKA and Ca^2+^ signaling pathways is involved in mitochondrial biogenesis [17], [18], [33], [34] and improves lipid oxidation at rest and during submaximal exercise [11], [35]. Recent studies have identified critical transcription factors mediating the effect of induction of these pathways at the cellular level. Costford et al. compared the effects of exercise training *in vivo* to that of the PFI cocktail and found that mitochondrial content and NAMPT, an enzyme involved in the SIRT1-PGC-1α mitochondrial biogenesis pathway, gene expression were increased in both conditions [22]. PGC-1α and PPARδ are major players in the cAMP/PKA and Ca^2+^ signaling cascade leading to mitochondrial biogenesis [33], [34], [36], [37]. We found that pharmacological activation of cAMP/PKA and Ca^2+^ signaling pathways did in fact induce PGC-1α and PPARδ gene expression, although not significantly due to variation, and mitochondrial content. In line with increased mitochondrial content, the myotubes displayed higher lipid oxidation for both oleate and palmitate and increased the activity of the rate-limiting enzyme of the β-oxidation pathway, β-HAD. Recent data from Koves et al. [38] suggests that the mismatch between β-oxidation and complete mitochondrial oxidation generates intermediate products that are critical to the development of insulin resistance. Our results show that PFI-activated myotubes exhibited higher rates of complete oxidation to CO_2_, reflecting enhanced coupling of mitochondrial β-oxidation to the electron transport chain, which was accompanied by an improvement in insulin responsiveness for glycogen storage. By increasing mitochondrial content and the lipid oxidation machinery efficiency, PFI treatment couples β-oxidation to complete mitochondrial oxidation. Notably, while the complete oxidation to CO_2_ is similar for both palmitate and oleate, the ratio of CO_2_∶ASM is higher for oleate, thus indicating lower ASMs. This is likely due to the fact that acutely oleate is more readily stored than palmitate [39] and therefore produces less ASMs.

These *ex vivo* results with the novel PFI treatment are similar to the effects of exercise training in skeletal muscle, which is also known to activate the cAMP/PKA and Ca^2+^ signaling pathways, along with various others. For example, Tarnoplosky et al found that 7 weeks of aerobic training in lean young men and women increased the number of lipid droplets and changed their intracellular distribution within the muscle. Transmission electron micrograph revealed a reorganization of lipid droplets along the myofibrillar apparatus, as well as an increase in their physical contact with mitochondria [35]. Those functional changes in intramyocellular lipid (IMCL) distribution were associated with increased total mitochondria area and capacity for lipid oxidation during exercise. In the present study, the PFI treatment increased the number of lipid droplets along the myofibrillar apparatus, as well as in the perinuclear region where the triacylglycerols are thought to be packaged [40]. Interestingly, these changes in lipid droplet distribution were closely associated with changes in lipid droplet protein (ADRP and TIP-47) gene expression. In a recent study by Prats et al., ADRP and TIP-47 were identified as the main lipid droplet proteins associated with intramyocellular triacylglycerol (IMTG) in rat skeletal muscle [41]. They showed that epinephrine or electrical stimulation of isolated single muscle fibers reduced IMTG content and translocated HSL to ADRP and IMTG. We also observed a higher lipolytic response to isoproterenol after activation of cAMP/PKA and Ca^2+^ signaling pathways in cultured myotubes. This enhanced lipolytic capacity was related to higher expression of the lipases HSL and CGI-58. HSL is thought to be a major lipolytic enzyme in skeletal muscle [42], [43], [44]. CGI-58 has been identified as a potent co-activator of ATGL, another lipase responsible for triacylglycerol breakdown in murine and human adipose [45], [46], [47] and skeletal muscle tissue [48]. Based on our results, CGI-58 could also play an important role in the regulation of IMTG breakdown in human skeletal muscle.

Lastly, another important facet of skeletal muscle lipid metabolism is lipid storage capacity. In a recent report, Schenk and Horowitz have shown that a single bout of exercise (90 minutes at 65% of VO_2_ peak) is sufficient to improve lipid storage capacity in skeletal muscle by partitioning more fatty acids toward triacylglycerol synthesis [49]. This increased IMTG storage capacity was associated with higher protein levels of GPAT and DGAT1 in human skeletal muscle. We found increased total lipid and triacylglycerol synthesis in response to the PFI treatment. This was accompanied by a higher gene expression of rate-limiting enzymes of lipid synthesis including GPAT and DGAT1. While we did not measure the identity of all the lipid species other than triacylglycerol, diacylglycerol and phospholipid, several studies support the idea that increased skeletal muscle lipid storage and/or DGAT1 activity is protective against lipid-induced insulin resistance by channeling more fatty acids into the triacylglycerol pool and reducing the production of bioactive “lipotoxic” compounds altering insulin signaling and glucose metabolism [49], [50]; thus, the increased storage of triacylglycerol rather than diacylglycerol or phospholipid is sufficient to support this notion.

Together with structural changes in lipid droplet morphology and functional changes in lipid metabolism, the PFI treatment increased basal- and insulin-stimulated glycogen storage capacity (i.e. non-oxidative glucose disposal). Insulin-mediated glucose transport into muscle is the rate-controlling step for insulin-stimulated muscle glycogen synthesis [3]. As such, insulin-stimulated glycogen storage is a relevant marker of insulin responsiveness. Furthermore, myotubes have a high basal expression of Glut1 and Glut4 and thus a high basal glucose uptake; therefore, it is not surprising that we did not observe a detectable difference in basal or insulin-stimulated glucose uptake between the control and the PFI treatment. In this model, changes in oxidative capacity, lipid storage capacity and lipolytic capacity are each closely associated with improvement in insulin responsiveness. Interestingly, these components of lipid metabolism are all rate-limiting steps in IMTG turnover. The reorganization of IMTG may channel lipids into oxidation and away from the generation of “lipotoxic” intermediates.

It is important to note that other effects of forskolin, ionomycin and/or their combination could indirectly influence the outcomes we measured. While we did not measure the activity of signaling pathways known to be activated by forskolin or ionomycin, evidence from similar studies on the effects of palmitate and other fatty acids on these signaling pathways [19], [20], as well as our in-depth examination of the functional metabolic outcomes, suggests that these agents in combination did indeed lead to the effects presented in this paper.

In summary, we have demonstrated that the activation of cAMP/PKA and Ca^2+^ signaling pathways with a novel pharmacological treatment results in a change in the intracellular distribution of lipid droplets, functional changes in lipid synthesis, lipolysis and lipid oxidation, as well as insulin-mediated glycogen storage. This model provides important insights into the regulation of lipid metabolism and insulin responsiveness in a cell culture system. We also characterize a more physiologically relevant *ex vivo* model of muscle lipid metabolism, which can be genetically manipulated and intensely studied to further dissect complex relationships between the organization of lipid droplets, mitochondrial oxidative capacity and insulin responsiveness, especially in obesity and disease states such as type 2 diabetes.

## Supporting Information

Table S1 **Oligonucleotide sequences for primer/probe sets used for qRT-PCR.** ND1 and LPL were used as indicators of mtDNA content. ND1, NADH dehydrogenase subunit 1; LPL, lipoprotein lipase. For all other gene expression assays the ribosomal phosphoprotein large P0 gene RPLP0 was used as the internal control. SREBP-1b, sterol responsive element binding protein-1, b isoform; FASN, fatty acid synthase; GPAT, glycerol-3-phosphate acyl-transferase; DGAT1, acyl CoA:diacylglycerol acyltransferase; ADRP, adipocyte differentiation-related protein; TIP47, tail-interacting protein 47; HSL, hormone sensitive lipase; ATGL, patatin-like phospholipase domain containing 2; CGI58, comparative gene identification 58; PGC1α, peroxisome proliferator-activated receptor gamma, coactivator 1 alpha; PPARδ, peroxisome proliferator-activated receptor delta; RPLP0, ribosomal protein, large, P0.
(DOC)
