# Supplementary material for: Remodeling Lipid Metabolism and Improving Insulin Responsiveness in Human Primary Myotubes
Source: PLoS One. 2011 Jul 8;6(7):e21068. doi: 10.1371/journal.pone.0021068 (PMC3132732; doi:10.1371/journal.pone.0021068)
Supplement: Table S1 — Oligonucleotide sequences for primer/probe sets used for qRT-PCR. ND1 and LPL were used as indicators of mtDNA content. ND1, NADH dehydrogenase subunit 1; LPL, lipoprotein lipase. For all other gene expression assays the ribosomal phosphoprotein large P0 gene RPLP0 was used as the internal control. SREBP-1b, sterol responsive element binding protein-1, b isoform; FASN, fatty acid synthase; GPAT, glycerol-3-phosphate acyl-transferase; DGAT1, acyl CoA:diacylglycerol acyltransferase; ADRP, adipocyte differentiation-related protein; TIP47, tail-interacting protein 47; HSL, hormone sensitive lipase; ATGL, patatin-like phospholipase domain containing 2; CGI58, comparative gene identification 58; PGC1α, peroxisome proliferator-activated receptor gamma, coactivator 1 alpha; PPARδ, peroxisome proliferator-activated receptor delta; RPLP0, ribosomal protein, large, P0. (DOC) [file pone.0021068.s001.doc]

**Table S1. Oligonucleotide sequences for primer/probe sets used for qRT-PCR**

| Gene | **Accession number** | Forward primer | Probe | Reverse primer |
| --- | --- | --- | --- | --- |

| ND1 |  | CCCTAAAACCCGCCACATCT | CCATCACCCTCTACATCACCGCCC | GAGCGATGGTGAGAGCTAAGGT |
| --- | --- | --- | --- | --- |
| LPL | NM_00237.2 | CGAGTCGTCTTTCTCCTGATGAT | ACATTCACCAGAGGGTC | TTCTGGATTCCAATGCTTCGA |
| SREBP-1b | NM_004176.4 | GGAGAACCTAAGTCTGCGCACT | CTGTCCACAAAAGCAAATCTCTGAAGGATCTG | TCCCTCCACTGCCACAGG |
| FASN | [NM_004014](http://www.ncbi.nlm.nih.gov/nuccore/NM_004104.4) | TATGCTTCTTCGTGCAGCAGTT | AGCGCCTCCAGCACCCTGTTGT | GCTGCCACACGCTCCTCTAG |
| GPAT | NM_020918.4 | ACTCCTTGGGCCTTTGCTG | CCTACAGCTCTGCTGCCATCTTTGTTCA | TTCTGGAACAGGACCACTGAAGT |
| DGAT1 | NM_012079 | CGTGAGCTACCCGGACAATC | ACCTACCGCGATCTCTACTACTTCCTCTTCGC | AAAGTTGAGCTCGTAGCACAAGG |
| ADRP | NM_001122.2 | GCATTGGATATGATGATACTGATGAGTC | CCACTGTGCTGAGCACATTGAGTCACGT | GCGGGCAATTGCAAGAGT |
| TIP47 | NM_005817.3 | AGCACCCAGGTGACAGTGG | CACACTGGGCTGCTGTACCGGTTCTT | CATGCTGGCCACACGGT |
| HSL | NM_005357.2 | ACGCTGCATAAGGGATGCTT | AGTTCACGCCTGCCATCCGGC | CCTGTCTCGTTGCGTTTGTAGT |
| ATGL | NM_020376.2 | CCACGGCGCTGGTCA | TTGGCACCAGCCTCACCCAGG | GGGCCTCTTTAGATACCTCAATGA |
| CGI58 | [NM_006016](http://www.ncbi.nlm.nih.gov/nuccore/NM_016006.4) | GGAGAGAGGTCAGGATGGCTAA | CTCCCCACATGGTGCCCTACGTCTATATC | AACATCTTCTCTTCAGCTTCTTTAAGG |
| PGC1α | NM_013261.3 | TGCTGAAGAGGGAAAGTGAGCGATTAGTTGA | CATGTAGAATTGGCAGGTGGAA | AGGTGAAAGTGTAATACTGTTGGTTGA |
| PPARδ | NM_006238.3 | TCTACAATGCCTACCTGAAAAACTTC | ACATGACCAAAAAGAAGGCCCGCAG | GGCTTTGCCGGTGAGGAT |
| RPLP0 | NM_001002 | CCATTCTATCATCAACGGGTACAA | TCTCCACAGACAAGGCCAGGACTCG | AGCAAGTGGGAAGGTGTAATCC |

ND1 and LPL were used as indicators of mtDNA content. ND1, NADH dehydrogenase subunit 1; LPL, lipoprotein lipase. For all other gene expression assays the ribosomal phosphoprotein large P0 gene RPLP0 was used as the internal control. SREBP-1b, sterol responsive element binding protein-1, b isoform; FASN, fatty acid synthase; GPAT, glycerol-3-phosphate acyl-transferase; DGAT1, acyl CoA:diacylglycerol acyltransferase; ADRP, adipocyte differentiation-related protein; TIP47, tail-interacting protein 47; HSL, hormone sensitive lipase; ATGL, patatin-like phospholipase domain containing 2; CGI58, comparative gene identification 58; PGC1α, peroxisome proliferator-activated receptor gamma, coactivator 1 alpha; PPARδ, peroxisome proliferator-activated receptor delta; RPLP0, ribosomal protein, large, P0.
